# Assessing Peripheral Focus in Myopes and Non-myopes: Introducing “Depth-of-refraction”

**DOI:** 10.1007/s44402-026-00023-5

**Published:** 2026-03-06

**Authors:** Charlie Börjeson, Shrilekha Vedhakrishnan, Anna-Caisa Söderberg, Anna Lindskoog Pettersson, Peter Unsbo, Linda Lundström

**Affiliations:** 1 Department of Applied Physics, KTH Royal Institute of Technology, Stockholm, Sweden; 2 Department of Health Sciences, Mid Sweden University, Östersund, Sweden; 3 Department of Clinical Neuroscience, Karolinska Institutet, Solna, Sweden

**Keywords:** Depth-of-focus, Emmetropia, Multifocality, Myopia, Peripheral image quality, Objective refraction

## Abstract

**Purpose:**

Peripheral image quality is of high relevance to myopia research, yet peripheral refraction is difficult to define due to aberrations affecting the depth-of-focus. This study investigated the peripheral image quality (±25° horizontal field) using three different image quality metrics, with added sphero-cylindrical wavefronts to find the best correction.

**Methods:**

Nineteen adults (9 myopes, 10 emmetropes) and 33 children (5 myopes, 28 non-myopes) were measured using a dual-angle wavefront aberrometer as part of the Stockholm Myopia Study. The optical image quality was calculated from the wavefronts, for 10,000 different sphero-cylindrical corrections around the 2nd-order Zernike refraction, to find the best correction as well as the range of corrections with similar image quality (“depth-of-refraction”).

**Results:**

Overall, the peripheral best focus was not distinct, with a large depth-of-refraction. Emmetropes/non-myopes had larger peripheral depth-of-refraction than myopes (mean values of 2.69 and 1.74 D, respectively (Strehl ratio metric)). For some subjects, this span of corrections was of a multifocal character. The prevalence of multifocality depended on the image quality metric but was generally more common in emmetropes/non-myopes than in myopes.

**Conclusions:**

The peripheral visual field does not always have a clear best focus and can show multifocal properties in some individuals, with different corrections yielding similar image quality. As emmetropes/non-myopes had more multifocality and larger depth-of-refraction than myopes, this indicates that inherent peripheral optical properties can play a role in myopia development.

Key points

- Peripheral best focus is not distinct: there is a range of corrections (“depth-of-refraction”) with similar image quality, often spanning multiple dioptres.
- On average, non-myopic subjects had larger depth-of-refraction in the periphery than myopes.
- More non-myopes than myopes were found to have persistent multifocality in the periphery (25° horizontal field), i.e., two separate corrections with similar image quality.

## Introduction

Peripheral image quality has been identified as an important factor in myopia development. In animal studies, peripheral blur has been shown to affect myopic growth [1, 2], and relative peripheral refraction (RPR) can alter refractive development [3–6], with hyperopic RPR inducing myopia and myopic RPR preventing myopic growth. Consequently, inducing peripheral myopic defocus has become a primary design feature in optical myopia control treatments [7–11]. However, higher-order aberrations in the periphery often make determining the peripheral refraction difficult.

For foveal refraction, subjective refraction remains the gold standard since it accounts for the entire visual system (optical and neural) of the subject [12]. On the other hand, objective refraction only uses the optical properties of the eye to estimate the subjective refraction, though sometimes in combination with models of the neural processing. Therefore, final estimation depends on measurement technique, computational choices and neural assumptions, and as a result, the calculated refraction varies [13, 14]. Moreover, most objective techniques were developed for foveal measurements [15], where higher-order aberrations are relatively low in magnitude. For peripheral refraction, the stronger aberrations will lower the image quality overall and increase the depth-of-focus, thereby creating further issues for defining the best focus and determining refraction, both objectively and subjectively [14].

The aim of the current study was to investigate objective peripheral refraction, specifically by exploring the “best focus”. Three image quality metrics assessing different aspects of the image were used for this analysis. Data were used from peripheral wavefront measurements, and the retinal image quality was simulated for a broad range of sphero-cylindrical corrections. It was found that there was not one distinct best focus, but rather a spread of corrections that gave similar image quality (over a range of a few dioptres), sometimes in the form of multifocality. Furthermore, this study investigated whether this “depth-of-refraction” and multifocality differed between emmetropes/non-myopes and myopes, and between adults and children.

## Methods

This investigation utilised data from two previous studies within the Stockholm Myopia Study, both conducted by the KTH Visual Optics group in Stockholm, Sweden. The studies were approved by the Swedish Ethical Review Authority (Reference Nos. 2023-01477-01 and 2021-04523) and were conducted in accordance with the Declaration of Helsinki.

The first study is an ongoing longitudinal study on the role of peripheral image quality during accommodation on refractive development in children [16]. Wavefront data and refraction for 33 children aged 6–11 years old were obtained from the baseline measurements. The children were classified as myopic if their spherical equivalent refraction (SER) was ≤−0.5 D and non-myopic if SER > −0.5 D. The SER was obtained using cycloplegic autorefraction with cyclopentolate 1% and the Wave Analyzer Medica 700 (Essilor Instruments, essilorinstrumentsusa.com).

The second study is a case-control study on the peripheral image quality during accommodation in adult myopes and emmetropes [17]. Nine myopes (habitual correction ≤ −1.5 D) and ten emmetropes (|SER| ≤ 0.5 D, verified by autorefraction with the Wave Analyzer Medica 700) aged 20–37 years old were recruited as part of this second study. The best corrected visual acuity was at least 1.0 decimal equivalent (6/6).

**Fig. 1:**
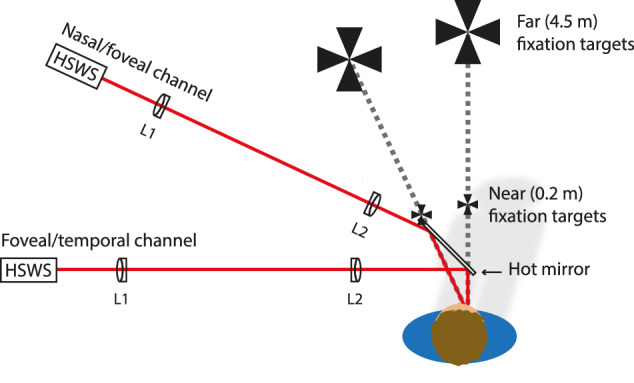
Top view of the dual-angle aberrometer setup (simplified) [18]. The system contained two measurement channels, one that measures the foveal wavefront error and one that measures 25° out in the horizontal visual field. If the subject looks at one of the left targets, the peripheral measurements will be in the temporal visual field (assuming measurements on the right eye). Conversely, if the subject looks at one of the right targets, the peripheral measurements will be in the nasal visual field. Two different target distances were used: 4.5 m (0.22 D) and 0.2 m (5 D). The hot mirror allows for open-field binocular fixation. HSWS Hartmann-Shack wavefront sensor. L1 + L2 telescope relay lenses.

Both studies used the same open-field dual-angle wavefront aberrometer (for more details, see Romashchenko et al. [18]) to capture information on the foveal and peripheral image quality of the right eye. The instrument has two measurement channels, allowing simultaneous measurements of two visual field angles: foveal +25° nasal visual field, or foveal +25° temporal visual field, depending on subject fixation, see Fig. 1. The instrument collects wavefronts continuously, with an acquisition rate of ~6 Hz per channel: for example, a 5 s scan would result in 30 saved foveal-peripheral wavefront pairs. Measurements were made for two accommodation demands: 0.22 (4.5 m) and 5 D (0.2 m). For both accommodation demands, measurements were performed in both the nasal and the temporal visual field. Hence, wavefront data were available for four peripheral field-distance cases: nasal 4.5 m, nasal 0.2 m, temporal 4.5 m and temporal 0.2 m. Wavefronts were captured and saved continuously during about 2–10 s of stable fixation for each field-distance case, repeated at least three times. If there were substantial acquisition losses, e.g., due to sudden head movements, blinks or reflections, then additional repetitions were performed. Subjects who wore a habitual refractive correction kept this on during the wavefront measurements.

### Preprocessing of Data

The raw wavefront images were assessed manually to filter out low-quality images, i.e., images with strong corneal reflections, misalignments, partially obscuring eyelids, blinks, quick movements or other kinds of degradations. From the remaining images, wavefronts were reconstructed with Zernike polynomials. The reconstructed wavefronts were then converted from 830 nm (measurement wavelength) to 550 nm. Mean spheres (*M*) calculated with up to 6th-order Zernike coefficients were then assessed to allow for exclusion of wavefronts with reconstruction errors or unstable accommodation. Additionally, only wavefronts that had a time-matching foveal/peripheral image were included, as this ensured that the accommodation of the subject was known for the peripheral images. After filtering, there were an average of 91 foveal-peripheral wavefront pairs per field-distance case for each subject (due to the continuous capturing), yielding a total of 723 ± 127 wavefronts per subject (average ± standard deviation (SD)).

### Image quality simulations

Foveal and peripheral image quality were analysed using three different image quality metrics. The primary metric was the Strehl ratio (STR) of the point spread function (PSF), as it is a widely used image quality metric and closely related to the visual STR, which has previously been used successfully for objective refraction foveally [13, 19, 20]. Furthermore, two secondary image metrics were used for comparison with the STR results: correlation width (CW) of the PSF [13] and the MTFa. These three metrics were chosen as they do not require knowledge of the neural processing (which has not been well studied in the periphery) and measure different aspects of the image quality: STR measures the contrast in the PSF (peak intensity relative to the diffraction limited case); CW measures the compactness of the PSF and MTFa measures the overall contrast transfer for grating objects. Furthermore, both STR and CW have shown a good correlation with subjective refraction [13].

The image quality was calculated for a broad range of sphero-cylindrical corrections to create 3-dimensional (3D) maps showing how the image quality varied depending upon the correction expressed in power vectors, i.e., *M*, *J*_0_ and *J*_45_ [21]. However, although calculating the image quality metrics for a single wavefront is quick, doing so for a broad range of added defocus and astigmatism values can become computationally demanding. Moreover, there were around 700 wavefronts per subject, further increasing the computational demand by several orders of magnitude. To speed up the computations, a search algorithm was implemented to concentrate the computation around corrections with better image quality. In essence, the search started from the 2nd-order Zernike refraction [*M*, *J*_0_, *J*_45_] in the 3D refraction space and then expanded the “shell” around the starting point in intervals of 0.1 D. The algorithm expanded the shell stepwise, each step expanding from the point in the shell with the highest image quality. This way, the “volume” of calculated refractions grew only in the regions with higher image quality, skipping regions where the image quality was low. The algorithm was run separately for each image quality metric. A pseudocode version of the search algorithm can be found in Appendix A.

**Fig. 2:**
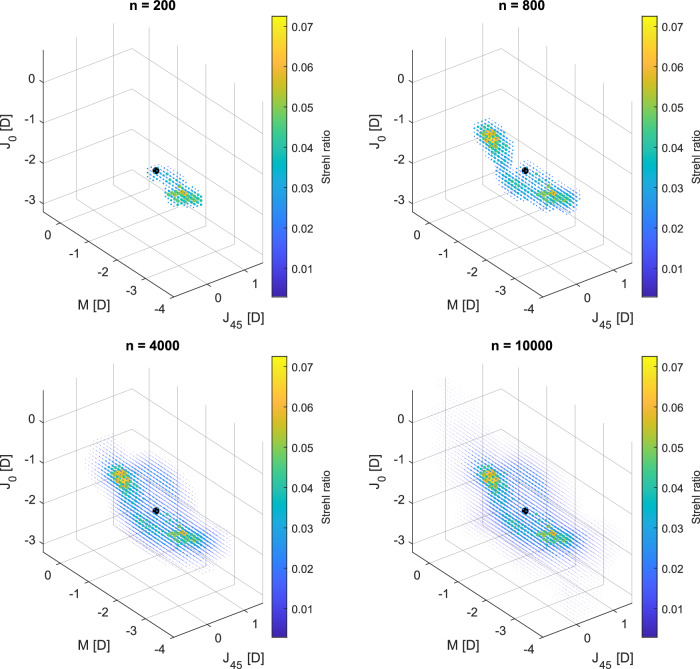
Example of how the search algorithm works to generate a 3D refraction map from a peripheral wavefront measurement. The black dot denotes the starting point, i.e., the 2nd-order Zernike refraction. The algorithm calculates the image quality (in this case, the Strehl ratio (STR)) of this point’s neighbours (intervals of 0.1 D). It then identifies the point in the shell with the best image quality, from where it will continue searching for new neighbours. This creates a growing volume of calculated STRs, with the shell expanding stepwise where the STR is highest. The figure shows the visited points in this volume and their corresponding STR for the first 200 points, 800 points, 4000 points and 10,000 points. Larger dots and warmer colours indicate higher STRs. *M*, *J*_0_ and *J*_45_ indicate the refraction in vectors.

The algorithm searched until 10,000 refractions, and their corresponding image quality had been calculated, as this was generally enough to find the whole refraction range, without wasting too many computing resources. Figure 2 shows an example of how the algorithm searched, with snapshots after 200, 800, 4000 and 10,000 visited points.

The refraction maps were calculated for both natural pupils and from wavefronts scaled to a 4 mm pupil diameter (maximum diameter, elliptical shape remaining) [22]. Furthermore, the Stiles-Crawford effect was accounted for, using the formula described by Atchison et al. [23].

### Depth-of-refraction and multifocality assessment

**Fig. 3:**
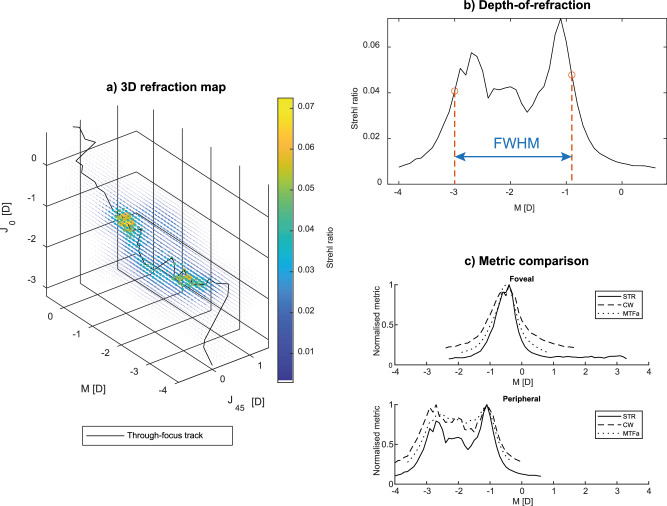
Depth-of-refraction and multifocality analysis. **a** The 3D refraction map shows the calculated Strehl ratios (STR) for 10,000 different refractive corrections for one peripheral wavefront for one subject (far fixation, same wavefront as Fig. 2). Two distinct regions with higher STR can be seen, i.e., there is multifocality. A through-focus track (with varied *J*_0_ and *J*_45_) identifies the best refraction for each *M*-value. **b** The through-focus track is plotted separately as a depth-of-refraction curve (in terms of mean sphere). The full-width-at-half-maximum (FWHM) shows the depth-of-refraction value, for which the STR is within half of the best value. **c** Foveal and peripheral depth-of-refraction curves, for all three image quality metrics (same peripheral wavefront as in (**a**, **b**)). Double peaks can be seen for all three image quality metrics, though weaker for MTFa: the relative prominence was 0.36 for STR, 0.35 for CW and 0.12 for MTFa. *M*, *J*_0_ and *J*_45_ indicate the refraction in vectors. CW correlation width, MTFa area under the one-dimensional modulation transfer function.

It was evident from the 3D refraction maps that there was often not a clear best focus, but rather a range of refractions with similar image quality. To quantify this, the refraction range for which image quality was not degraded by more than half of the best value was defined as the “depth-of-refraction”. In terms of mean sphere, this was done by wandering in the M-direction within the 3D refraction map and identifying the highest image quality value in each “*M*-slice” (see Fig. 3a). This through-focus track was saved along with the corresponding *M*-values. From this through-focus track, the depth-of-refraction was calculated as the full-width-at-half-maximum (FWHM) (see Fig. 3b). Median depth-of-refraction values for each field-distance case were then calculated for each subject.

In the depth-of-refraction analysis, it was clear that some wavefronts had separate refraction regions with similar image quality, i.e., multifocality. This was particularly clear for STR and CW, but could also be seen for MTFa (see Fig. 3c). To investigate possible multifocality, peak fitting was performed on the depth-of-refraction curves. A minimum peak separation of 0.75 D was required, and if more than one peak was found, then the prominence of the second-highest peak was calculated (i.e., the second peak’s height above the inter-peak valley). The relative prominence (prominence of second-highest peak/maximum peak height) was calculated for each wavefront. Then, median relative prominence values for each subject and field-distance case were calculated. A field-distance case was marked as multifocal when the median relative prominence was at least 20%. Subjects with no multifocal field-distance cases were classified as having *no multifocality*; subjects having one field-distance case with multifocality were classified as having *partial multifocality* and subjects with 2–4 field-distance cases with multifocality were classified as having *persistent multifocality*. Only subjects for which there was wavefront data for all four field-distance cases were used in the multifocality analysis.

### Depth-of-Focus

In addition to the depth-of-refraction and multifocality assessment, a similar analysis using the pure depth-of-focus (no astigmatic correction) was made. In the 3D refraction map, this corresponds to a straight line along the *M*-axis (*J*_0_ = 0 and *J*_45_ = 0). This refraction region was rarely computed in the peripheral depth-of-refraction assessment (due to off-axis astigmatism resulting in larger values of *J*_0_ in the periphery) and therefore had to be calculated separately. One hundred points (=10 dioptres) along the *M*-axis were calculated, with the starting point being the 2nd-order Zernike mean sphere refraction. The FWHM of this through-focus track is the depth-of-focus. Median depth-of-focus values for each field-distance case were calculated for each subject.

This depth-of-focus corresponds to the direct depth-of-focus experienced by the eye, since real objects only create spherical wavefronts, not sphero-cylindrical ones. Since the eye’s astigmatism is not accounted for, there is off-axis astigmatism present in the periphery, which could give rise to two line-foci, largely affecting the characteristics of the depth-of-focus. Multifocality was not investigated in the depth-of-focus, as the aim of this paper was to investigate the characteristics of the best focus in terms of sphero-cylindrical correction.

### Group Comparisons

Wilcoxon tests were used to evaluate if there were significant differences in depth-of-refraction and depth-of-focus between myopes and emmetropes/non-myopes, with adults and children being treated separately. Tests were performed both for natural pupils and for 4 mm pupils, using the values calculated with STR, for all field-distance cases combined. In total, eight tests were performed. To account for the multiple tests, the Bonferroni correction was used to achieve a new, corrected significance level. With *α* = 0.05, the corrected significance level was *α*_corr_ = 0.05/8 = 0.00625. Note that when scaling the pupils to 4 mm, measurements smaller than 4 mm were removed, resulting in fewer field-distance cases for some subjects. To compare the different image quality metrics, average depth-of-refraction and depth-of-focus values were calculated for each subject (mean of the field-distance cases).

## Results

In total, wavefront data from 52 subjects were included in the study. In the adult group, 19 subjects were included, of whom nine were myopic (mean SER ± SD–2.55 ± 0.50 D) and 10 emmetropic (0.16 ± 0.14 D). The mean age (±SD) of the adults was 26.3 ± 4.5 years. Three of the myopic subjects wore spectacles as their habitual correction, while six wore contact lenses.

Five of the 33 children were myopes (−1.53 ± 1.11 D). Two of these children wore spectacles, while the rest were uncorrected due to them only being mildly myopic. The remaining 28 non-myopic children had a mean SER of +0.99 ± 0.60 D, meaning that some of them could be classified as currently hypermetropic (one was wearing spectacles for hypermetropia). However, since the children were young and still undergoing emmetropisation, no further distinction between the non-myopes was made for the purposes of this article. The mean age of the children was 8.2 ± 1.3 years.

For one myopic and one non-myopic child, there were no data for some field-distance cases for natural pupils, due to a large number of failed wavefront captures. Furthermore, as some subjects had small natural pupils, especially during accommodation, there were others lacking field-distance cases for the 4 mm analysis.

### Peripheral Best Focus and Refraction

The 3D refraction maps showed that there was often no clear best focus in the periphery, since similar image quality could be found for a broad range of sphero-cylindrical corrections. In terms of the mean sphere, the range of similar corrections (depth-of-refraction) could span several dioptres. It was also evident that for some subjects, there were *separated* regions in the 3D refraction map with similar image quality, i.e., there was inherent multifocality. This was most prominent for STR and CW. This multifocality also meant that the best correction could vary by a couple of dioptres between two consecutive wavefront captures on the same subject. Consequently, peripheral refraction can be very difficult to define accurately, particularly in subjects with larger depth-of-refraction and/or multifocality.

### Peripheral Depth-of-refraction and Depth-of-focus

**Fig. 4:**
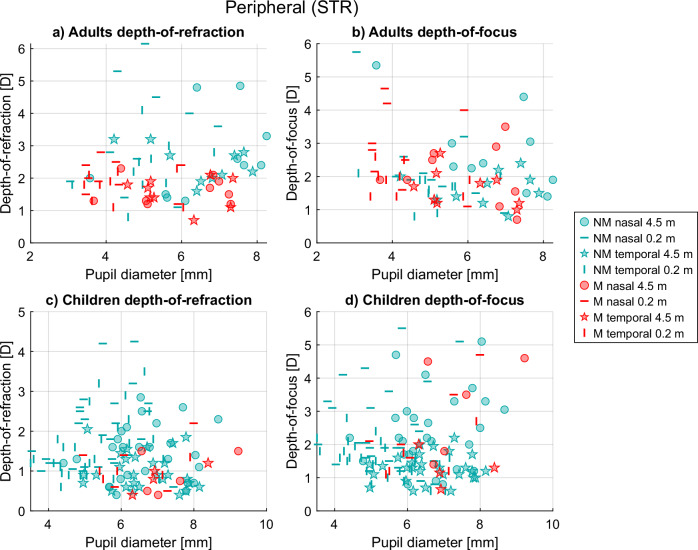
Peripheral depth-of-refraction and depth-of-focus (median values for each subject and field-distance case) for natural pupils (STR metric). Non-myopes (NM) and myopes (M) are indicated by cyan and red markers, respectively. Each subject was measured both 25° temporally and nasally for two accommodation levels (4.5 m (0.22 D) and 0.2 m (5 D)). **a** Depth-of-refraction for adults. There was a significant difference between myopes and non-myopes ($p=6\times \,{{10}^{-5} < \alpha }_{{{\rm{corr}}}}$). **b** Depth-of-focus for adults. There was no significant difference between myopes and non-myopes. **c** Depth-of-refraction for children. There was a difference between non-myopes and myopes ($p=0.01$), but it was not significant under Bonferroni correction. **d** Depth-of-focus for children. There was no significant difference between myopes and non-myopes. STR Strehl ratio.

The peripheral depth-of-refraction and depth-of-focus (STR metric) for all subjects and field-distance cases can be found in Fig. 4. Figures for the other image quality metrics, as well as for the foveal data, are available in Appendix B.

For the adults, the peripheral depth-of-refraction for natural pupils was significantly different between the myopes and emmetropes ($p=6\times {10}^{-5}\,( < {\alpha }_{{{\rm{corr}}}})$) (see Fig. 4a). The myopes had an overall smaller depth-of-refraction than the emmetropes, with mean values of 1.74  ± 0.48 and 2.69 ± 1.20 D, respectively (STR metric). As can also be seen in the figure, the depth-of-refraction was similar between the 25° nasal and temporal fields, and it was also similar between the far and near measurements, despite the smaller pupil size during the near measurements. Notably, the mean *foveal* depth-of-refraction was almost identical between the adult myopes and emmetropes, with both groups having mean values of 1.06 D (STR metric).

The same trend in peripheral depth-of-refraction was observed in the children, with the few myopes being in the lower end of the range (see Fig. 4c). The difference between the non-myopic and myopic children was not significant under Bonferroni correction, although still notable ($p=0.01$). Compared to the adults, the children generally had smaller peripheral depth-of-refraction, with myopes having a mean value of 1.01 ± 0.47 D and non-myopes 1.50 ± 0.80 D (STR metric). The foveal depths-of-refraction for the children were 0.71 and 0.82 D for the myopes and non-myopes, respectively.

Contrary to depth-of-refraction, no significant difference in peripheral depth-of-focus was found between the refractive groups, either in adults (Fig. 4b) or in children (Fig. 4d). Furthermore, when scaling the pupils to a 4 mm diameter, no significant difference was seen between the refractive groups in terms of depth-of-refraction, nor in depth-of-focus (STR metric).

**Fig. 5:**
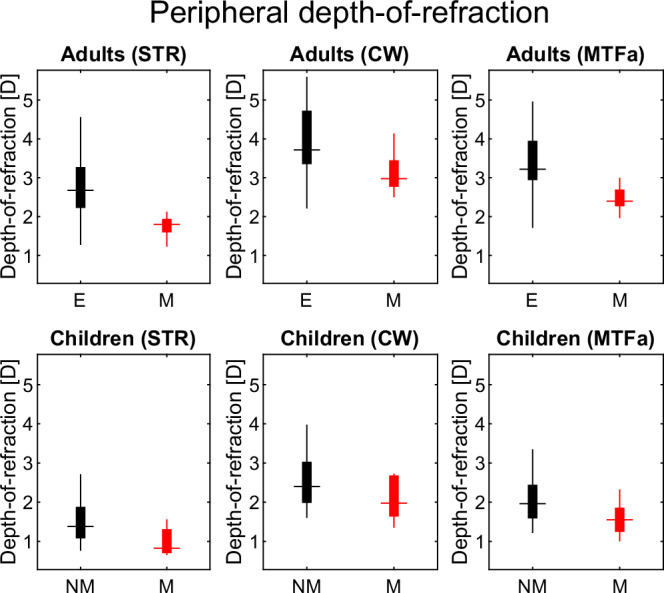
Peripheral depth-of-refraction boxplots for the adults and children (average values for each subject), using all three image quality metrics and natural pupils. The bottom and top of the boxes indicate the 25th and 75th percentiles, the horizontal bars indicate the median values, and the ends of the vertical whiskers indicate the minimum and maximum values. STR Strehl ratio, CW correlation width, MTFa area under the one-dimensional modulation transfer function, Emmetropes (E) and non-myopes (NM) are shown in black, and myopes (M) are shown in red.

Figure 5 shows the peripheral depth-of-refraction for adults and children, separated by refractive groups, as calculated from all three image quality metrics. For all three metrics, myopes had smaller depth-of-refraction than emmetropes/non-myopes, for both adults and children. Of the three metrics, STR gave the smallest depth-of-refraction values, whereas CW gave the largest. This is also reflected in a comparison between the peripheral 2nd-order Zernike refraction and the depth-of-refraction ranges: the 2nd-order mean sphere was outside of the depth-of-refraction range in 12% of wavefronts for STR, 0.3% of wavefronts for CW and 1.6% of wavefronts for MTFa.

### Peripheral multifocality

**Fig. 6:**
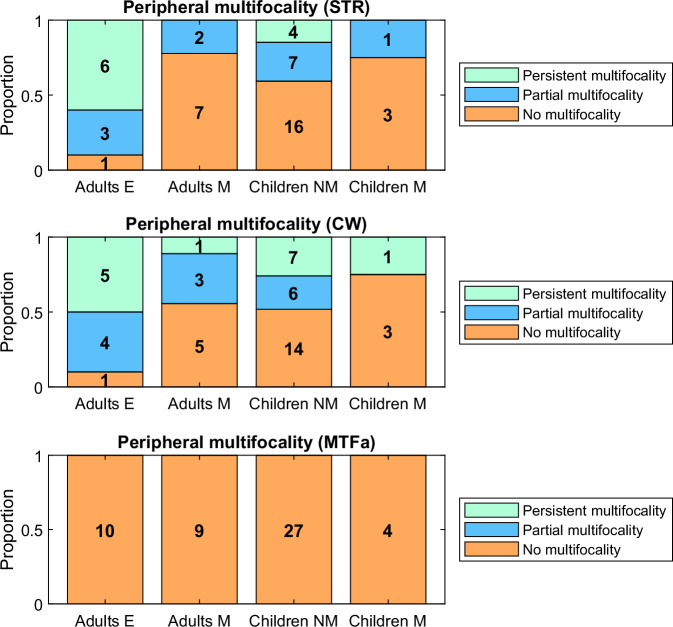
Amount of multifocality found in the different subgroups, for natural pupils. E emmetropes, M myopes, NM non-myopes. No multifocality: zero field-distance cases with multifocality. Partial multifocality: one field-distance case with multifocality. Persistent multifocality: at least two field-distance cases with multifocality. STR Strehl ratio, CW correlation width, MTFa area under the one-dimensional modulation transfer function.

For STR and CW, multifocality was more common in the emmetropes/non-myopes than in the myopes (see Fig. 6). For STR, no myopes were found to have persistent multifocality (i.e., at least two field-distance cases with median relative prominence ≥ 20%), but among the emmetropes/non-myopes, six out of 10 adults and four out of 27 children had persistent multifocality. CW showed similar results as STR, although with slightly higher rates of multifocality. For MTFa, the threshold was too high, and no multifocality was found in any subject.

When scaling the pupils to 4 mm diameter (STR metric), the multifocality was lost in almost all subjects: no children showed any multifocality, and of the adults, only two had some multifocality (one emmetrope and one myope), with the rest having no multifocality.

## Discussion

This study investigated how peripheral image quality depended on sphero-cylindrical corrections in 33 children and 19 adults. Often, there was a broad range of corrections with similar image quality, making peripheral refraction very difficult to define accurately. Sometimes there were two separated regions with higher image quality, i.e., inherent multifocality in terms of sphero-cylindrical refraction in the periphery.

These results demonstrate that refraction in the periphery can be difficult to define, as multiple sphero-cylindrical corrections gave similar image quality. Oftentimes, peripheral refraction is calculated directly from the 2nd-order Zernike coefficients, but this does not always coincide with the point having the best image quality. Furthermore, the point with the best image quality can vary markedly between measurements due to random fluctuations, particularly if the subject has multifocality. Thus, it can be challenging to compare studies on peripheral refraction and RPR, since all instruments that sum the refraction into a single value will have to choose how to determine the refraction. Further studies are needed to understand how the output of commercial auto-refractors relates to the 3D refraction maps described here.

**Fig. 7:**
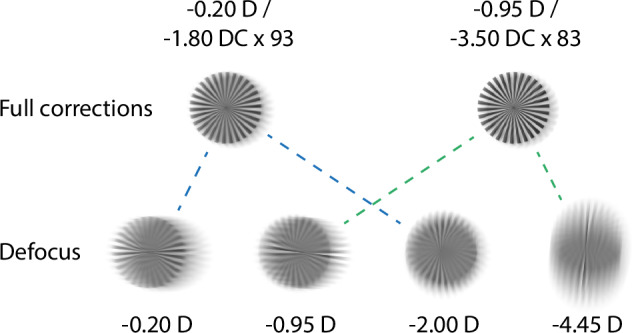
Simulations of a Siemens star (2° visual angle) as imaged in the 25° nasal visual field of a subject with inherent multifocality (same wavefront as in Figs. 2 and 3). The two best sphero-cylindrical corrections yield the images shown in the top row. Split into their meridional powers, the through-focus images shown on the bottom row are obtained, where the two sets of astigmatic line foci can be seen clearly. D dioptre sphere, DC dioptre cylinder.

The kind of multifocality described here can be difficult to visualise, as it is not simply defocus, but a combination of defocus and astigmatism. One way to illustrate the multifocality is to think in terms of line foci. With one single sphero-cylindrical refractive error, i.e., no multifocality, there would be two image planes for the two line foci from astigmatism. With multifocality, there would be four image planes (see Fig. 7).

It was found that more emmetropes/non-myopes had inherent multifocality in the periphery than myopes, when using natural pupils (STR and CW metrics). Furthermore, on average, myopes had a smaller depth-of-refraction than the emmetropes/non-myopes. However, there was no significant difference in depth-of-focus between the myopes and emmetropes/non-myopes. Since sphero-cylindrical wavefront corrections were used, this indicates that both multifocality and depth-of-refraction are a result of higher-order aberrations. Additionally, the multifocality disappeared when scaling the pupils to 4 mm, further suggesting the involvement of higher-order aberrations (as they are greatly diminished at 4 mm due to their strong pupil dependence). The differences in depth-of-refraction and multifocality between emmetropes/non-myopes and myopes indicate that peripheral higher order aberrations can play a role in myopia development. Future studies on these phenomena are needed to identify if any particular aberrations, or combinations of aberrations, are particularly involved in shaping the depth-of-refraction and multifocality.

Even though both high multifocality and larger depth-of-refraction were associated with emmetropia/non-myopia, there was no apparent direct correlation between multifocality and depth-of-refraction, i.e., a large depth-of-refraction did not necessarily mean multifocality, or vice versa. This indicates that the two are separate phenomena, although they are both likely caused by higher-order aberrations. No significant correlation was observed between depth-of-refraction and pupil size for natural pupils (Fig. 4). On an individual level, though, the depth-of-refraction generally got smaller when scaled to 4 mm pupils.

There was no significant difference in central refraction between the myopic adults and myopic children, but there was a significant difference in refraction between the emmetropic adults and non-myopic children ($p=5\times {10}^{-4}$). This was because the mean sphere limits for the refractive groups differed between the children and adults: the study on children recruited a wider selection of participants, regardless of refraction, whereas the adult study aimed to recruit specifically myopes and emmetropes, with some refractive separation between the two groups. In the present study, the adult and child populations were treated as two distinct refractive groups in order to perform similar analyses for all data. As some of the children are likely to become myopic in the coming years, one should be careful when drawing conclusions from the group of non-myopic children, since their future refractive development is yet unknown. Furthermore, it should be noted that there were fewer myopes than non-myopes in both age groups, particularly for the children. Ideally, there would have been an even distribution of myopes and non-myopes in both age groups.

In this study, the STR (which measures contrast in the PSF) was used as the primary image quality metric, as the visual STR has previously been used successfully for foveal measurements [13, 19, 20]. Additionally, CW and MTFa were used as two complementary image quality metrics. CW measures the compactness of the PSF, whereas MTFa measures the area under the one-dimensional modulation transfer function. Of the three metrics, STR was the most sensitive to defocus: the depth-of-refraction was smaller than for CW and MTFa, and the 2nd-order refraction more often lay outside of the depth-of-refraction range compared with CW and MTFa. In the multifocality assessment, a 20% relative prominence was chosen as the threshold for multifocality. This threshold was fitting for STR and CW, and the automatic classification found similar rates of multifocality among the subjects as found with subjective examination. However, the MTFa depth-of-refraction curves, though also displaying double peaks, generally had much lower relative prominences than for STR and CW (see the example in Fig. 3c), resulting in no subject having >20% median relative prominence with MTFa. This shows that the choice of threshold and metric can have a large impact on the multifocality classification.

Unlike the visual STR, no neurological effects were accounted for apart from the Stiles–Crawford effect when calculating the image quality, since not enough is known about neural processing in the periphery. However, to verify that the multifocality found in the present study would be plausible for the eye to detect, PSFs were filtered down to 10 cycles per degree (cpd) and 5 cpd for two of the multifocal subjects. The multifocality was still present at 10 cpd, although lost at 5 cpd (STR metric). As this is somewhere between the resolution and detection thresholds [24–27], it seems probable that the multifocality can be detected by the eye. It should also be noted that the formula used to compensate for the Stiles–Crawford effect was derived from foveal measurements, and the Stiles–Crawford effect has been shown to be even larger in the periphery than at the fovea [28]. As the multifocality likely comes from higher-order aberrations and therefore primarily from the edges of the pupil, it is possible that the detected multifocality by the eye is less prominent than the results indicate.

## Conclusion

In this study, peripheral wavefront data were analysed from 33 children and 19 adults. Optical image quality was simulated under a broad range of sphero-cylindrical corrections. It was found that the range with similar image quality, i.e., the depth-of-refraction, could span several dioptres, making peripheral refraction difficult to define. Additionally, the peripheral depth-of-refraction was larger in emmetropes/non-myopes than in myopes, but there was no significant difference in terms of depth-of-focus. Furthermore, some subjects had inherent multifocality (STR and CW metrics) in the periphery, i.e., two different sphero-cylindrical corrections that yielded very similar image quality, with corrections between the two yielding worse image quality. Multifocality was more prevalent amongst emmetropes/non-myopes than myopes. These results indicate that higher-order aberrations in the periphery, affecting multifocality and depth-of-refraction, could play a role in myopia development.

## Figures and Tables

**Fig. 8:**
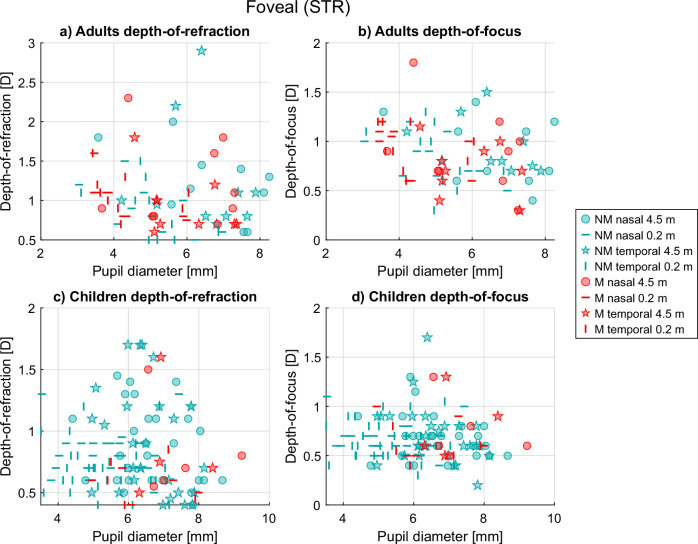
Foveal depth-of-refraction and depth-of-focus (median values for each subject and field-distance case) for natural pupils (STR metric). Non-myopes (NM) and myopes (M) are indicated by cyan and red markers, respectively. Each subject was measured for four field-distance cases: foveal + nasal (4.5 and 0.2 m) and foveal + temporal (4.5 and 0.2 m). **a** Depth-of-refraction for adults. **b** Depth-of-focus for adults. **c** Depth-of-refraction for children. **d** Depth-of-focus for children. STR Strehl ratio.

**Fig. 9:**
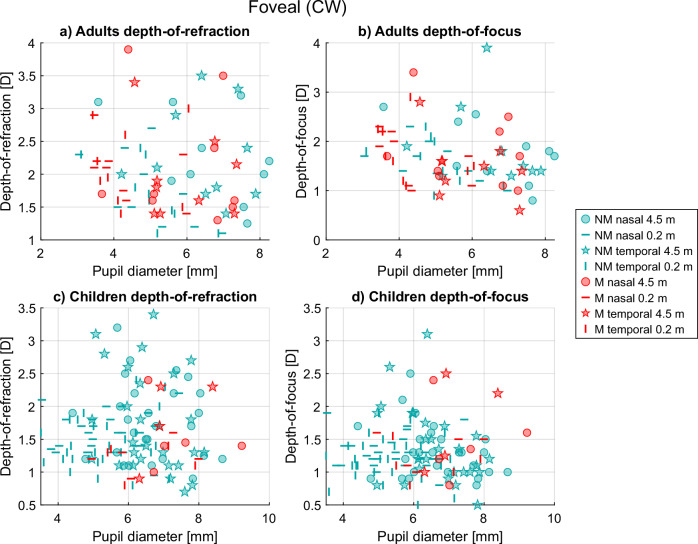
Foveal depth-of-refraction and depth-of-focus (median values for each subject and field-distance case) for natural pupils (CW metric). Non-myopes (NM) and myopes (M) are indicated by cyan and red markers, respectively. Each subject was measured for four field-distance cases: foveal + nasal (4.5 m and 0.2 m) and foveal + temporal (4.5 m and 0.2 m). **a** Depth-of-refraction for adults. **b** Depth-of-focus for adults. **c** Depth-of-refraction for children. **d** Depth-of-focus for children. CW correlation width.

**Fig. 10:**
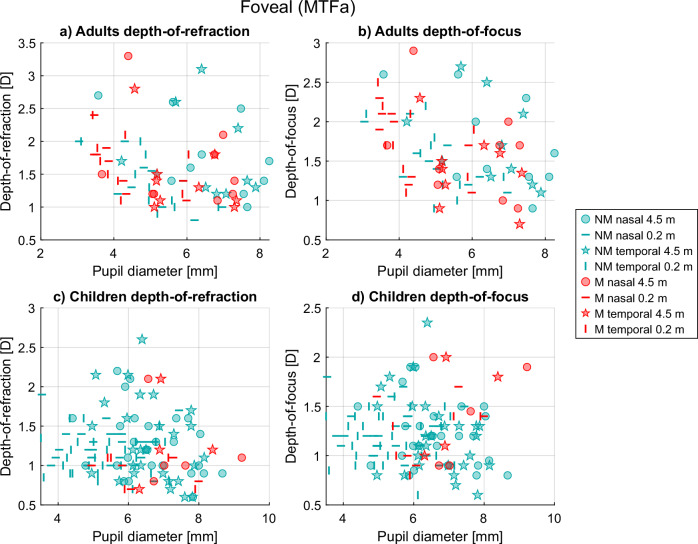
Foveal depth-of-refraction and depth-of-focus (median values for each subject and field-distance case) for natural pupils (MTFa metric). Non-myopes (NM) and myopes (*M*) are indicated by cyan and red markers, respectively. Each subject was measured for four field-distance cases: foveal + nasal (4.5 m and 0.2 m) and foveal + temporal (4.5 m and 0.2 m). **a** Depth-of-refraction for adults. **b** Depth-of-focus for adults. **c** Depth-of-refraction for children. **d** Depth-of-focus for children. MTFa area under the modulation transfer function.

**Fig. 11:**
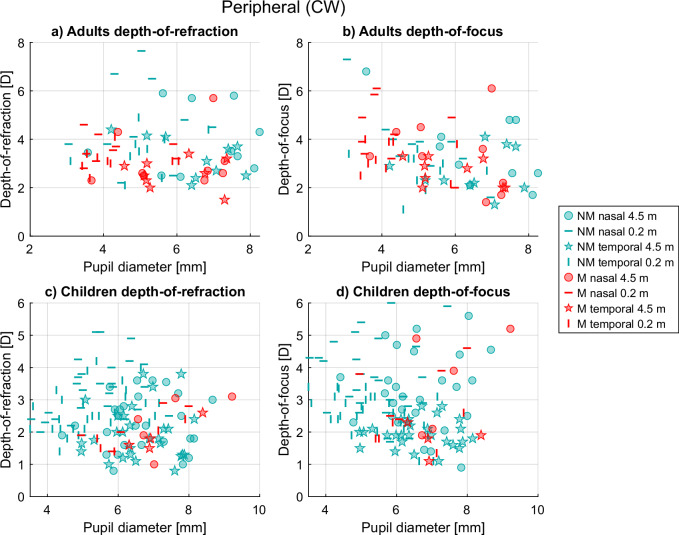
Peripheral depth-of-refraction and depth-of-focus (median values for each subject and field-distance case) for natural pupils (CW metric). Non-myopes (NM) and myopes (M) are indicated by cyan and red markers, respectively. Each subject was measured both 25° temporally and nasally for two accommodation levels (4.5 m (0.22 D) and 0.2 m (5 D)). **a** Depth-of-refraction for adults. **b** Depth-of-focus for adults. **c** Depth-of-refraction for children. **d** Depth-of-focus for children. CW correlation width.

**Fig. 12:**
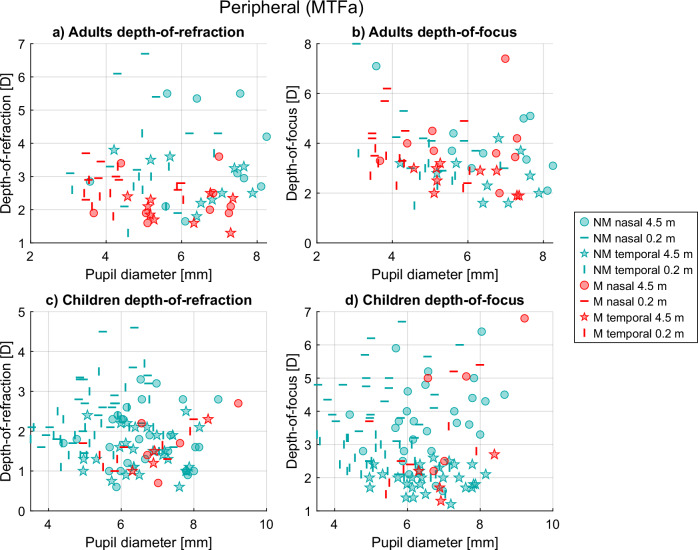
Peripheral depth-of-refraction and depth-of-focus (median values for each subject and field-distance case) for natural pupils (MTFa metric). Non-myopes (NM) and myopes (M) are indicated by cyan and red markers, respectively. Each subject was measured both 25° temporally and nasally for two accommodation levels (4.5 m (0.22 D) and 0.2 m (5 D)). **a** Depth-of-refraction for adults. **b** Depth-of-focus for adults. **c** Depth-of-refraction for children. **d** Depth-of-focus for children. MTFa area under the modulation transfer function.

## Data Availability

Data underlying the results may be obtained from the authors upon reasonable request.

## Appendix A

//higher image quality values = better image qualitystarting_guess = getZernikeRefraction(wavefront) //M, J0 and J45image_quality = getImageQuality(starting_guess, wavefront)calculated_points = [starting_guess, image_quality]points_to_search = [starting_guess, image_quality]**while** length(calculated_points) < 10000  best_guess = point with best IQ in points_to_search  remove best_guess from points_to_search  **for** all neighbours to the best_guess    **if** neighbour not in calculated_points     image_quality = getImageQuality(neighbour, wavefront)     add neighbour and image_quality to calculated_points     add neighbour and image_quality to points_to_searchreturn calculated_points

## Appendix B

Figures 8–12
